# Uncovering a role for METTL13 in malignant transformation of human hematopoietic stem cells and in the progression of pediatric leukemia

**DOI:** 10.21203/rs.3.rs-7652169/v1

**Published:** 2025-10-23

**Authors:** Sabina Enlund, Chae-Eun Lim, Isabella Hoang, Sonali Joshi, Amanda Ramilo Amor, Cecilia Thomsson, Indranil Sinha, Shahrzad Shirazi Fard, Anna Nilsson, Ola Hermanson, Qingfei Jiang, Frida Holm

**Affiliations:** 1 Deparment of Women’s and Children’s Health, Division of Pediatric Oncology and Surgery, Karolinska Institutet, 171 77 Stockholm, Sweden; 2 Division of Regenerative Medicine, Department of Medicine, Moores Cancer Center, University of California, San Diego, La Jolla, CA, USA, Moores Cancer Center, La Jolla, CA 92037, USA; 3 Department of Laboratory Medicine, Karolinska Institutet, 141 52 Huddinge, Sweden; 4 Department of Neuroscience, Karolinska Institutet, 171 77 Stockholm, Sweden

## Abstract

Post-transcriptional RNA modifications, such as N6-methyladenosine (m6A) methylation and adenosine to inosine (A-to-I) editing, are critical regulators of hematopoietic stem cell (HSC) self-renewal and differentiation, yet their precise contributions to malignant transformation are not fully elucidated. In this study, we uncovered the epitranscriptomic landscape caused by knockdown of genes from the methyltransferase (METTL)-family in hematopoietic stem and progenitor cells (HSPCs). We identified both converging and distinct roles of METTL3 and METTL14, known members of the m6A writer complex, as well as orphan gene METTL13. Notably, METTL13 was uniquely upregulated by adenosine deaminase acting on RNA 1 (ADAR1) overexpression, while other METTL genes were downregulated. Knockdown of METTL13 altered the expression of multiple genes involved in oncogenic development in HSPCs. Furthermore, METTL13 was associated with a high-risk profile in pediatric T-cell acute lymphoblastic leukemia (T-ALL), and functional studies confirmed that METTL13 is required for T-ALL cell proliferation and survival both *in vitro* and *in vivo*. Collectively, our results indicate a previously unrecognized, oncogenic role for METTL13 in pre-leukemic transformation and T-ALL pathogenesis.

## Introduction

Epitranscriptomic alterations, which comprise numerous post-transcriptional RNA modifications, have emerged as important drivers of disease development and cancer progression (1). RNA modifications shape the cellular response to environmental cues, by regulating cell survival, differentiation and migration. Aberrant activity of these RNA modifications can contribute to malignant transformation and has been linked to a wide variety of cancers (2).

Among RNA modifications, N6-methyladenosine (m6A) methylation and adenosine to inosine (A-to-I) editing are the most abundant, both targeting adenosines on mRNA to alter its fate and function (3). A-to-I editing is achieved by adenosine deaminase acting on RNA 1 (ADAR1) and has been studied for its role in regulating innate immunity and maintaining hematopoietic stem cells (HSCs) (4–6). Aberrant A-to-I RNA editing impairs stem cell fitness and promotes cancer development (7–9). Malignant overexpression of ADAR1 has been reported in over 20 types of cancers, including pediatric T-cell acute lymphoblastic leukemia (T-ALL) (7, 10).

RNA methylation by the m6A complex is a dynamic process mediated by a large network of proteins, many of which are not completely characterized (3, 11–13). Methylation is accomplished by a group of methyltransferases (METTLs), predominantly METTL3 and METTL14, which make up the core of the writer complex (14). This complex further interacts with proteins like WT1 associated protein (WTAP) and METTL16 for specific recruitment of RNA. The reversible capacity of m6A modifications is achieved by two known erasers, fat mass and obesity-associated (FTO) and demethylase AlkB homolog 5 (ALKBH5) (13, 15–17). Different readers, such as YTH-containing proteins and heterogeneous nuclear ribonucleoproteins (HNRNPs), recognize and interpret m6A sites on mRNA transcripts, ultimately determining the transcript fate and downstream effects (13, 18–20). Modifications by the m6A complex affect many aspects of mRNA metabolism, including mRNA stability, splicing and translation (21–24). Abnormal activity of m6A methylation has been associated with many diseases, including neurological and autoimmune disorders (2, 16, 25–30). Recent studies have also concluded a critical role for m6A methylation in maintaining stem cell-like capacities of leukemia stem cells (LSCs), which can potentiate therapy resistance and tumor metastasis (31).

Many of m6A functions overlap with those of ADAR1. Both highly regulated m6A methylation and A-to-I editing are required for HSC self-renewal and differentiation (4–6, 8, 32–37), and malignant activity of these modifications can promote the development of both solid tumors and hematological cancers^,^ (37–41). Although these two types of RNA modifications are known to regulate HSC fitness separately, little is known about how these pathways interact to fine-tune cellular functions in normal HSCs and in the transformation to LSCs. Both m6A and A-to-I RNA modifications target adenosine bases, although it is still debated whether they compete for the exact same adenosine. A-to-I RNA editing occurs mainly in inverted Alu sequences enriched in intronic and 3’UTR regions, while m6A are located within highly conserved RRACH sequences within 3’UTR and exons (35, 42). Whether these two RNA-modifying processes functionally interact to promote malignant transformation and leukemia development is yet to be defined.

In this study, we investigated the role of the m6A complex in HSC maintenance and hematological malignancies, hypothesizing that METTL-family members coordinate with ADAR1 to drive malignant transformation. We report that overexpression of ADAR1 in hematopoietic stem and progenitor cells (HSPCs) suppressed most m6A-related genes, except for METTL13, an orphan METTL-family member whose function in HSCs and LSCs has not been reported. Knockdown of METTL3, METTL13 and METTL14 in HSPCs convergently disrupted many immune signaling and survival pathways, while METTL13 knockdown uniquely affected cancer-related pathways. Next, we investigated the role of METTL13 in pediatric acute lymphoblastic leukemia (ALL) and found that increased METTL13 expression correlated with a poor prognosis in both T-ALL and B-cell ALL (B-ALL). Loss of METTL13 led to arrested cell proliferation and reduced cell viability, both *in vivo* and *in vitro*. Knockdown of METTL13 in T-ALL cell suppressed pathways regulating proliferation and DNA-replication. Collectively, our findings reveal consequences of alterations to the METTL-family in disease development and recognizes an emerging role for METTL13 in hematological malignancies.

## Materials and Methods

### Cord blood and cell line handling

CD34^+^ human cord blood samples and peripheral blood mononuclear cells (PBMCs) were purchased from commercial vendors (StemCell Technology or AllCells) and stored in liquid nitrogen until ready for use. T-ALL cell lines (CEM, CVCL_0207; CUTTL1, CVCL_4966; MOLT-4, RRID:CVCL_A1BB; SUP-T1, RRID:CVCL_1714; and Jurkat, RRID:CVCL_0065) were purchased from ATCC and maintained in RMPI media (Gibco, 11875119) supplemented with 10% FBS (Gibco) and 1% penicillin-streptomycin (Gibco, 15140) at 37° C in 5% CO2.

### Lentiviral construct and transduction

To determine the function of members of the METTL-family, lentiviral knockdown in HSPCs and T-ALL cells was performed. Lentiviral construct of shRNAs targeting METTLs was purchased from VectorBuilder (Vector ID: VB240522–1552yyd). All lentivirus was tested by transducing HEK293T cells, and the knockdown efficiency and titers were assessed by FACS analysis of GFP signal and RT-qPCR of the target genes. Cord blood CD34^+^ cells were cultured in 96-well plate (5×10^5^ cells per well) containing StemPro (Life Technologies) media supplemented with human cytokines (IL-6, stem cell factor (SCF), Thrombopoietin (Tpo) and FLT-3, all from R&D Systems) for 2–3 days at lentivirus at a MOI of 100–200. T-ALL cell lines were cultured in 24-well plate or 6-well plate (5–10×10^5^ cells per well) in culture media with lentivirus at a MOI of 5–10. Cells were then collected for downstream analysis.

### Animal experiments and Flow Cytometry analysis

All mouse studies were conducted under protocols approved by the Institutional Animal Care and Use Committee (IACUC) of the University of California, San Diego and were in compliance with federal regulations regarding the care and use of laboratory animals: Public Law 99–158, the Health Research Extension Act, and Public Law 99–198, the Animal Welfare Act which is regulated by USDA, APHIS, CFR, Title 9, Parts 1, 2, and 3. Immunocompromised NSG-SGM3 mice were bred and maintained in the Sanford Consortium for Regenerative Medicine vivarium according to IACUC approved protocols of the University of California, San Diego. Neonatal mice of both sexes were used in the study. MOLT4 or SUP-T1 cells were injected intrahepatically into 2–3 days old neonatal NSG-SGM3 mice at 2.5–5×10^4^ cells per pup. Leukemic engraftment was quantified by FACS analysis-based peripheral blood screening of human CD45^+^ population starting from week 3 for every week until the engraftment reaches 10%. Mice were humanely sacrificed at 4–8 weeks, and cells were collected from hematological organs (bone marrow and spleen) for FACS analysis. Cells were resuspended in staining media (ice cold DPBS with 2% FBS), followed by blocking using FcR block (Cat 130–059-901, Miltenyi Biotech) for 15 minutes. CD45-APC cell surface antibody (Cat # 304037, Biolegend, San Diego, CA) was added a final dilution of 1:25 and incubated on ice for 30 min in the dark. DAPI solution was added before analysis to exclude dead cell debris. Flow analysis was performed on BD Aria Fusion, Aria II.

### Western Blot analysis

Cells were collected and washed three times with cold PBS and resuspended in RIPA buffer supplemented with protease and phosphatase inhibitors and quantified by BCA assay. Protein lysate (10 μg) was separated by 10% SDS–PAGE and transferred to PVDF membranes. The membrane was blocked in 5% BSA/20mM Tris-HCL for 30 min while rocking and probed with primary antibodies (METTL13 antibody, abcam, ab186002; beta-actin antibody, Millipore Sigma, A2228) overnight at 4 °C followed by incubation with HRP-conjugated secondary antibodies for 2 hours at room temperature. Signals were visualized using SuperSignal West Femto Substract (ThermoFish, #34096) on a ChemiDoc System (Bio-rad).

### RNA extraction and Quantitative Real-Time Polymerase Chain Reaction (RT-qPCR)

For validation of lentiviral knockdown, RNA was extracted and analyzed through RT-qPCR. RNA extraction was performed using RNeasy micro extraction kits (QIAGEN) following the manufacturer’s protocol. A DNase incubation step was included to digest any genomic DNA. RNA (100–1,000 ng) was converted to cDNA using the Super-Script III kit (ThermoFisher Scientific) according to the manufacturer’s recommended protocol. qRT-PCR was performed using SYBR GreenER Super Mix (Life Technologies) on BioRad CFX382 with 5 ng of template mRNA and 0.2 μM of each forward and reverse primer. Target mRNA was normalized to hypoxanthine phosphoribosyl transferase (HPRT) mRNA transcript levels and fold change was calculated via the delta-delta cycle threshold (CT) method. RT-qPCR primers are listed in supplemental table 5.

### Viability and proliferation analysis

To define effects of METTL13 knockdown on T-ALL cells, T-ALL cell lines were transduced with lentiviral scramble control or shRNA targeting shMETTL13 at a low MOI of 10–20 in culture media without pen/strep. After 2–3 days, cells were plated in 96-well plates at 1 ×10^5^ cells per well. At each time point, cell number and viability were determined by trypan blue assay.

### RNA-sequencing following lentiviral knockdown

The RNA-sequencing dataset transduced with ADAR1 overexpression in HSPCs was available from previous studies (BioProject: PRJNA319866) (6). For RNA-sequencing of METTL knockdown in HSPCs and T-ALL, samples with RNA integrity numbers (RIN) ≥7 were proceeded for bulk RNA-sequencing. RNA-sequencing of HSPCs transduced with METTL3, METTL5, METTL9, METTL13 and METTL14 knockdown was performed using 50–100 ng of RNA by Scripps Research on SMARTer seq system, with paired-end 150 reads, at 50 million reads per sample. Obtained reads were aligned using STAR two-pass alignment method and the reference genome GRCh38.84 together with the corresponding GFT file, to generate transcriptome-coordinate based BAM files as described in a previous study (10). Raw counts were obtained through STAR, using the ENCODE STAR-RSEM pipeline generating the numbers of reads aligned to each gene. Transcripts per million (TPM) values were calculated over the total collapsed exonic regions for each gene. RNA-sequencing of T-ALL cells transduced with METTL13 knockdown was performed using 100 ng of RNA by Novogene on NovaSeq X Plus sequencing system with paired-end 150 reads. Raw counts were generated from BAM files together with the provided GTF file in R (version 4.3.1) using FeatureCount from the Rsubread package (version 2.16.1), with the pipeline set to unstranded and paired end reads. Raw count data was normalized in R (version 4.3.1) to TPM using the mRNA expression transformation guideline provided by the GDC bioinformatics pipeline.

### Publicly available RNA-sequencing downloading and normalization

RNA-sequencing data from pediatric T-ALL patients was obtained from the publicly available data generated by the TARGET initiative. The RNA-sequencing dataset (.BAM) were acquired via the GDC Data Portal (https://portal.gdc.cancer.gov/projects/TARGET-ALL-P2). A total of 190 bone marrow-derived T-ALL samples (192 from time of diagnosis, 18 after relapse, non-longitudinal) and 78 bone marrow-derived B-ALL samples (39 from time of diagnosis, 39 after relapse, longitudinal) were included. Sample inclusion was based on the sample type (bone marrow), disease status (diagnosis and relapse) as well as MLL status (positive or negative, samples with status unknown were excluded). Raw counts were generated using the SeqMonk Mapped Sequence Data Analyzer tool (version 1.47.1). The RNA-seq quantitation pipeline was set to follow the subsequent criteria: transcript features were set to mRNA, library type was selected as opposing strand specific and transcript isoforms were merged. Raw count data from CD34^+^ cord blood was obtained from the publicly available data set found in the Gene Expression Omnibus (GEO) at GSE190269 (43). Raw count data was normalized in R (version 4.3.1) to TPM using the mRNA expression transformation guideline provided by the GDC bioinformatics pipeline.

### RNA quantification and functional enrichment

Subsequent analysis was based on normalized RNA-sequencing data (TPM), generated from raw counts as described above. PCA and heatmaps were created using Qlucore Omics Explorer (version 3.9). Significance for the heatmap was calculated using multi-group ANOVA (q<0.1, SD<0.05). Over-representation analysis (ORA) was performed by including all significantly differentially expressed genes (calculated using unpaired two-tailed t-test, p < 0.05) and generated in R (version 4.3.1), using packages ClusterProfiler (version 4.8.3), DOSE (version 3.28.2) and Enrichplot (version 1.20.3). For ORA, p-adjust method was set to BH and L2FC cutoff of 1. Gene set enrichment analysis (GSEA) was performed using GSEA software (version 4.3.2) provided by Broad Institute and UC San Diego (44, 45), utilizing human KEGG Legacy (version 2024.1.Hs), Reactome (version 2024.1.Hs) and Wiki Pathway (version 2024.1.Hs) gene sets. For GSEA, only protein-coding genes were included. For analysis of differentially expressed genes, genes with a L2FC = NA were excluded. Differentially expressed genes were obtained using unpaired two-tailed t-test between the different experimental conditions compared to the control group (p < 0.05).

### Statistical analysis

Statistical analyses were performed using ordinary one-way ANOVA of the mean of every condition against the control group with multiple comparisons (using normalized TPM values), with Dunnett correction or using unpaired two-tailed t-test when comparing normalized TPM values between two groups. Correlation was determined by Pearson correlation coefficients, two-tailed with 95% confidence interval. Analysis of cell viability and proliferation following *in vitro* analysis was performed using ordinary two-way ANOVA with multiple comparisons and analysis of cell engraftment *in vivo* was performed using Mann-Whitney test. The statistical analyses were performed using GraphPad Prism (v9) or R (version 4.3.1). Results are presented as the mean ± SEM. Significance was set to P < 0.05.

## Results

### ADAR1 overexpression suppressed members of the m6A complex in HSPCs.

**Figure 1.**
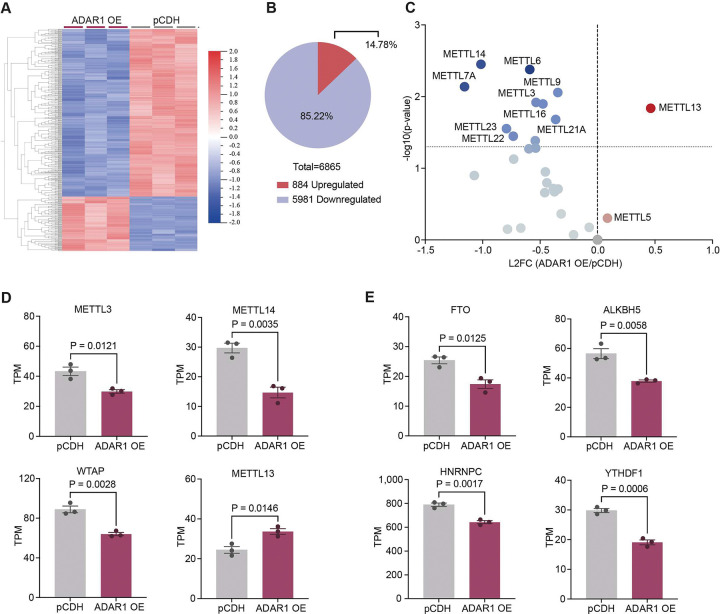
ADAR1 overexpression suppressed members of the m6A complex. A) Heat map of the top 500 differentially expressed genes in ADAR1 overexpressed human CD34^+^ HSPCs (ADAR1 OE, n=3) compared to the backbone control (pCDH, n=3). Created in Qlucore Omics Explorer, significance was calculated using unpaired two-tailed t-test, p<0.05. B) Distribution of differentially expressed genes in ADAR1 overexpressed HSPCs compared to the pCDH control. Significance was calculated using unpaired two-tailed t-test, p<0.05. C) Volcano plot of dysregulated genes from the METTL-family following ADAR1 overexpression. Significance was calculated usin unpaired two-tailed t-test, results are displayed as L2FC (ADAR OE/pCDH) and negative log10 p-value. D) Expression of genes from the m6A writer complex (METTL3, METTL14 and WTAP) and from the METTL-family (METTL13) in ADAR1 overexpressed cells compared to compared to the pCDH control. Significance was calculated using unpaired two-tailed t-test, results are displayed as TPM, mean ± SEM. E) Significantly differentially expressed m6A erasers FTO and ALKBH5 and readers HNRNPC and YTHDF1 in ADAR1 overexpressed cells compared to compared to the pCDH control. Significance was calculated using unpaired two-tailed t-test, results are displayed as TPM, mean ± SEM.

To investigate how ADAR1 regulates the m6A complex, we examined effects following lentiviral overexpression of ADAR1 in human CD34^+^ HSPCs (available at BioProject: PRJNA319866) (Fig. S1A). A total of 6 865 genes were significantly differentially expressed in ADAR1 overexpressed HSPCs compared to the backbone control (pCDH), with the majority (> 85%) of genes being downregulated (Fig. 1A, B, Supplemental table 1). By focusing on the m6A regulatory gene network, we found that overexpression of ADAR1 led to a downregulation of the main m6A writer complex (METTL3, METTL14 and WTAP) (28, 46, 47), m6A erasers (FTO and ALKBH5), and readers (HNRNPC and YTHDF1) (Fig. 1D, 1E). Other suppressed methyltransferase-like genes included METTL7A, a thiol methyltransferase(48); METTL9, a methyltransferase for 1-methylhistidine (49); and METTL16, which functions as both a non-conical nuclear m6A writer and cytosolic translation-initiator (50) (Figure SF1B). The only significantly upregulated gene from the METTL-family was METTL13. Together, these results indicate a potential suppressive relationship between ADAR1 and most METTL-family members, on a transcriptional level, except for METTL13. Since METTL3 and METTL14 comprise the main m6A writer complex, this suggests ADAR1 may suppress m6A modifications.

### Overlapping and distinct gene regulation programs by METTL3, METTL9, METTL13 and METTL14 in HSPCs.

**Figure 2.**
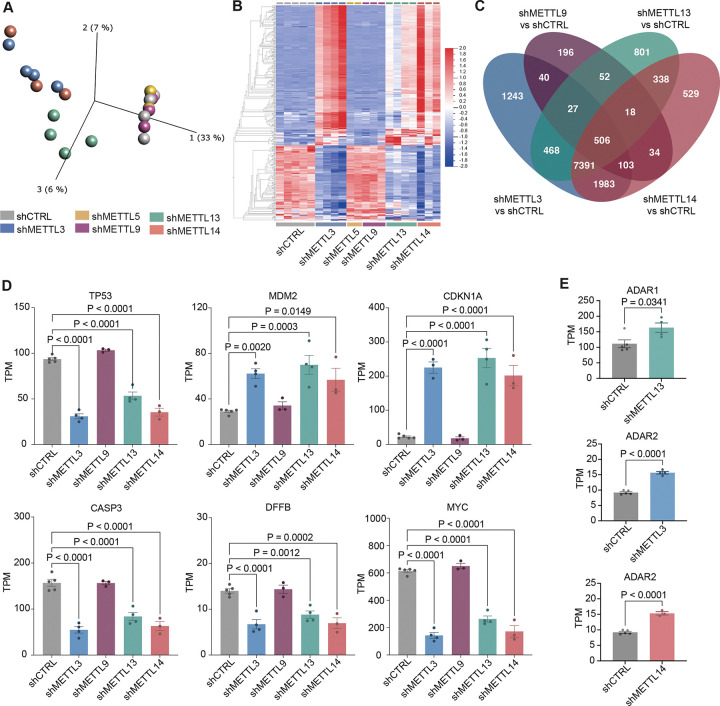
Overlapping and distinct gene regulation by METTL3, METTL9, METTL13 and METTL14 in HSPCs. A) PCA plot of METTL3 (shMETTL3, n=4), METTL5 (shMETTL5, n=2), METTL9 (shMETTL9, n=3), METTL13 (shMETTL13, n=4) and METTL14 (shMETTL14, n=3) knockdown in human CD34^+^ HSPCs compared to the backbone control (shCTRL, n=5). Created in Qlucore Omics Explorer. B) Heatmap of shMETTL3, shMETTL5, shMETTL9, shMETTL13 and shMETTL14 in HSPCs compared to shCTRL. Created in Qlucore Omics Explorer, significance was calculated using multi-group ANOVA, q<0.1, SD<0.05. C) Venn diagram of differentially expressed genes in shMETTL3, shMETTL9, shMETTL13 and shMETTL14 in HSPCs compared to shCTRL. Significance was calculated using unpaired two-tailed t-test, p<0.05, in each shMETTL compared to shCTRL. D) Dysregulated genes (TP53, MDM2, CDKN1A, CASP3, DFFB and c-MYC) in shMETTL3, shMETTL9, shMETTL13 and shMETTL14 compared to shCTRL. Significance was calculated using ordinary one-way ANOVA with multiple comparisons compared to shCTRL, as well as Dunnett correction, results are displayed as TPM, mean ± SEM. E) Differentially expressed genes from the ADAR family in shMETTL3, shMETTL13 and shMETTL14 compared to shCTRL. Significance was calculated using unpaired two-tailed t-test, results are displayed as TPM, mean ± SEM.

We systematically studied the gene expression landscape of several m6A family members affected by ADAR1 in human CD34^+^ HSPCs. Although the role of METTL3 and METTL14 are well established in HSC maintenance, METTL5, 9, and 13 have not been extensively studied in this context. To achieve this, lentiviral knockdown of METTL3, METTL5, METTL9, METTL13 and METTL14 was performed in CD34+ HSPCs (Fig. SF2A, SF2B). Principal component analysis (PCA) revealed three potential outlier samples, which were excluded in the subsequent analysis (Fig. SF2C). METTL3 and METTL14 knockdown conditions clustered together, separated from the control (Fig. 2A). Both shMETTL5 and shMETTL9 grouped together with the control, suggesting that METTL5 and METTL9 may have less effect on a transcriptional level, although knockdown efficacy requires improvement for further conclusions (Fig. 2A, 2B). Knockdown of METTL13 had similar effects on gene expression as METTL3 and METTL14, yet shMETTL13 HSPCs formed their own cluster away from the other METTLs in PCA, suggesting both overlapping and unique functions of METTL13 (Fig. 2A, 2B).

Next, we analyzed differentially expressed genes in each individual METTL knockdown condition compared to the control (Fig. 2C, Supplemental table 2). We excluded METTL5 due to low power after RNA quality control. Knockdown of METTL3, METTL13 and METTL14 shared 7 391 differentially expressed genes, while knockdown of METTL3 and METTL14 alone only shared 1 983 genes (Fig. SF2D). Our results suggest that the gene expression program of METTL13 substantially overlaps with that of m6A writer complex. To further investigate this, we carefully examined these overlapping target genes. Reduced expression of tumor suppressor TP53 and upregulation of its negative regulator MDM2 were reported (Fig. 2D) (51). Moreover, CDKN1A, an effector protein downstream of p53, was upregulated (52). Knockdown also suppressed apoptotic genes, including CASP3 and DFFB (53), and oncogenes such as c-MYC (54).

Since m6A modification was reported to promote ADAR1 activity (55), we examined if loss of METTL expression would alter ADAR1. We found that only knockdown of METTL13 led to increased ADAR1 expression (Fig. 2E). METTL3 and METTL14 knockdown caused an upregulation of ADAR2, which is mainly expressed and active in brain tissue, rather than the hematopoietic system (7). Taken together, these data suggest that METTL13 converges with METTL3 and METTL14 in regulating gene expression in HSPCs, while its distinct PCA clustering and unique gene expression profile indicates that METTL13 may have some unique properties.

### METTL3, METTL13 and METTL14 converged in regulating immune signaling while METTL13 caused distinct effects on apoptosis and p53 regulation.

**Figure 3.**
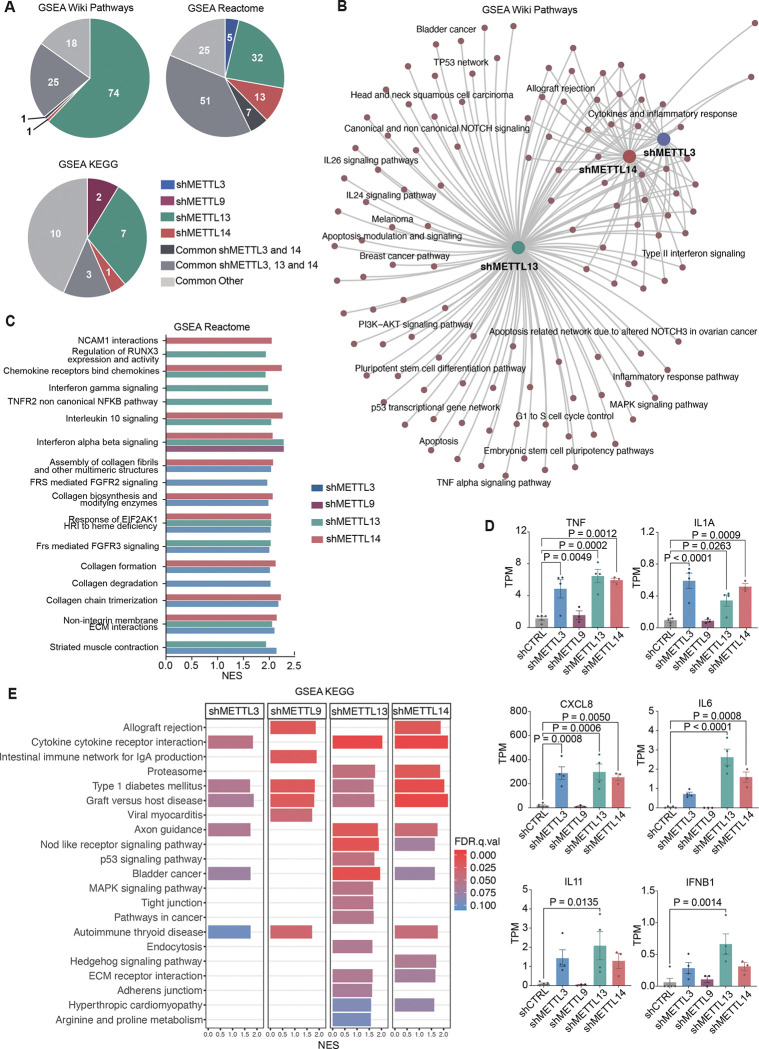
METTL3, METTL13 and METTL14 converged in regulating immune signaling, with many distinct pathways altered uniquely by METTL13. A) Distribution of significantly enriched pathways in each shMETTL compared to shCTRL generated through GSEA. Created in GSEA and MSigDB, FDR q<0.1, including only protein-coding genes, using three different gene set libraries (Wiki Pathways, Reactome and KEGG). Groups are based on which pathways are uniquely altered by shMETTL3 (n=4), shMETTL9 (n=3), shMETTL13 (n=4) or shMETTL14 (n=3), as well pathways altered in several conditions; the m6A writer complex (METTL3 and METTL14) with and without METTL13, as well as all other combined conditions, compared to shCTRL (n=5) in human CD34^+^ HSPCs. B) Network plot of GSEA Wiki Pathways in shMETTL3, shMETTL13 and shMETTL14 compared to shCTRL (FDR q<0.1). C) Top 10 significant GSEA Reactome pathways in shMETTL3, shMETTL9, shMETTL13 and shMETTL14 compared to shCTRL (FDR q<0.1). Results are displayed as normalized enrichment score (NES). D) Dysregulated genes involved in inflammatory signaling (TNF, IL1A, CXCL8, IL6, IL11 and IFNB1) in shMETTL3, shMETTL9, shMETTL13 and shMETTL14 compared to shCTRL. Significance was calculated using ordinary one-way ANOVA with multiple comparisons compared to shCTRL, as well as Dunnett correction, results are displayed as TPM, mean ± SEM. E) GSEA of shMETTL3, shMETTL9, shMETTL13 and shMETTL14 compared to shCTRL using KEGG legacy pathways (FDR q<0.1) Results are displayed as NES and FDR q-value.

To uncover signaling pathways regulated by METTL3, METTL9, METTL13 and METTL14 in HSPCs, we performed GSEA on each shMETTL compared to shCTRL, utilizing the WIKI Pathway, Reactome and KEGG libraries. Analysis revealed most unique altered pathways following METTL13 knockdown across all three libraries (Fig. 3A, SF3A, SF3B, SF3C). Most pathways that were altered by the core writer complex (METTL3 and METTL14) were also altered by METTL13, while METTL3 and METTL14 knockdown alone caused minimal overlap.

METTL13 knockdown exclusively enriched 74 Wiki Pathways, with 25 pathways altered in co-ordinance with METTL3 and METTL14 (Fig. 3B, SF3A). Distinct pathways included apoptosis and stem cell pluripotency, and several cancer-related gene sets, such as melanoma and breast cancer (Fig. 3B, SF3D). Top Reactome gene sets included several immune regulatory pathways, altered by METTL3, METTL9, METTL13 and METTL14 either together or separately (Fig. 3C). METTL3, METTL13 and METTL14 knockdown all increased expression levels pro-inflammatory cytokines such as TNF, IL1A and CXCL8 (Fig. 3D) (56–58). METTL13 knockdown uniquely increased expression of IL6, IL11 and IFNβ1a, which can inhibit cell cycle progression in hemopoietic malignances (59). GSEA also showed an enrichment of many inflammatory KEGG pathways after knockdown of the METTL-genes (Fig. 3E). Lastly, knockdown of METTL13 had the most exclusively dysregulated KEGG pathways, several crucial for malignant transformation, such as p53 signaling and pathways in cancer. Most altered pathways were either affected by the m6A writer complex together with METTL13 or by METTL13 alone, suggesting that METTL13 may be uniquely disposed to promote survival, proliferation and possibly oncogenic transformation of HSPCs.

### METTL13 knockdown altered pathways involved in malignant transformation.

**Figure 4.**
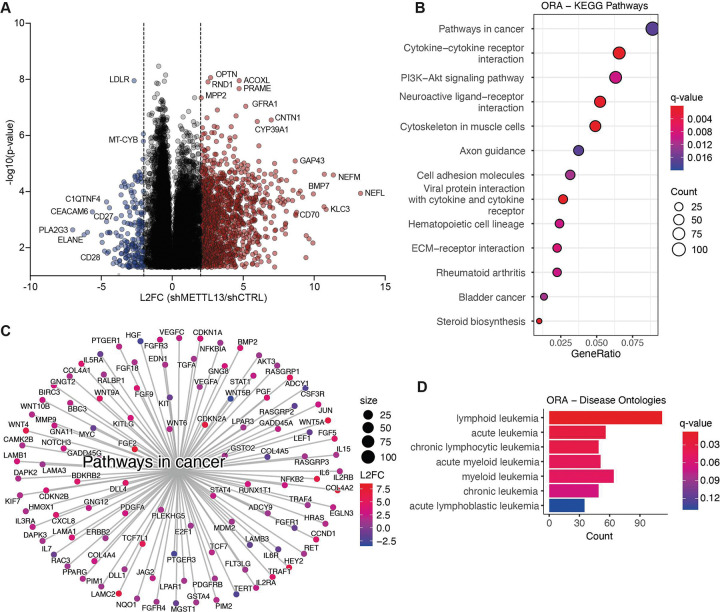
METTL13 knockdown caused dysregulation of pathways involved in malignant transformation. A) Volcano plot of differentially expressed genes following METTL13 knockdown (shMETTL13, n=4) compared to the control (shCTRL, n=5) in human CD34^+^ HSPCs. Significance was calculated using unpaired two-tailed t-test, results are displayed as L2FC and negative log10 p-value (p <0.05). B) ORA of the top enriched KEGG pathways in shMETTL13 compared to shCTRL. Created in R, with packages clusterprofiler and enrichPlot, statistics was set to: q.value <0.1, L2FC cutoff = 1, p-adjust method = BH. C) Network plot of the KEGG gene set pathways in cancer in shMETTL13 compared to shCTRL, nodes are colored by L2FC. D) ORA of disease ontologies focused on hematological malignances. Created in R, packages with clusterprofiler, DOSE and enrichplot, statistics were set to: q.value ≤ 0.1, L2FC cutoff = 1, p-adjust method = BH.

The unique gene expression program following METTL13 knockdown prompted us to define METTL13ś role in HSPCs. Inhibition of METTL13 led to altered expression of many genes involved in important cellular processes, including an upregulation of CD70, part of the TNF superfamily, which together with CD27 plays a role in LSC proliferation and survival (Fig. 4A, SF4A) (60). METTL13 knockdown also downregulated CEACAM6, which regulates tumor proliferation and migration through ERK-MAPK and PI3K-AKT signaling in several cancers, including lung and colon cancer (61).

ORA revealed dysregulation of several KEGG pathways following METTL13 knockdown, both inflammatory pathways, including TNF signaling and cytokine-cytokine receptor interaction, and pathways regulating cell survival, such as PI3K-Akt signaling (Fig. 4B). The pathway with the greatest number of dysregulated genes was pathways in cancer, many of which are part of the WNT and NOTCH signaling pathways, crucial for oncogenic progression (Fig. 4B, 4C). ORA of METTL13 knockdown also revealed involvement of METTL13 in several disease ontologies, many related to infection and inflammation, like encephalitis and pneumonia (Fig. SF4B). METTL13 knockdown also altered genes belonging to disease ontologies involved in hematological malignancies, mainly lymphoid leukemias and acute leukemias (Fig. 4D). This strengthens our hypothesis that METTL13 might have distinct molecular functions leading malignant alterations in HSPCs, potentially inducing pre-leukemic transformation.

### Upregulation of METTL13 was associated with a high-risk profile in T-ALL.

**Figure 5.**
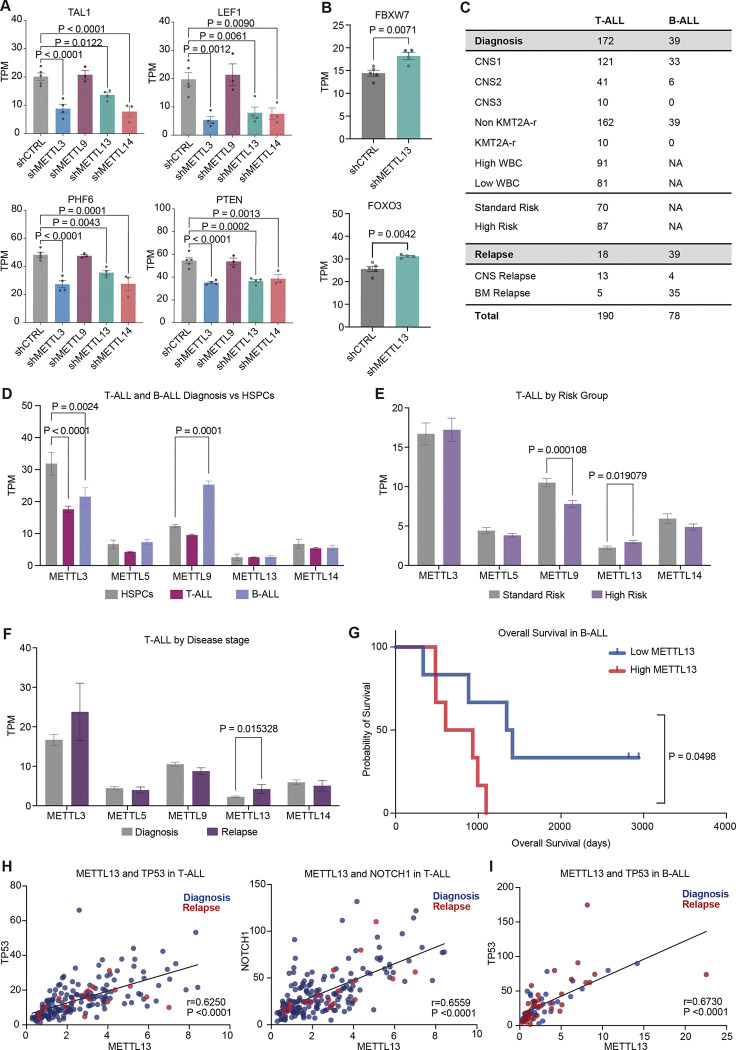
Upregulation of METTL13 associated with a high-T-ALL A) Dysregulated genes (TAL1, LEF1, PHF6 and PTEN) following METTL3 (n=4), METTL9, (n=3) METTL13 (n=4) and METTL14 (n=3) knockdown in human CD34^+^ HSPCs compared to the control (shCTRL, n=5). Significance was calculated using ordinary one-way ANOVA with multiple comparisons compared to shCTRL, as well as Dunnett correction, results are displayed as TPM, mean ± SEM. B) Uniquely dysregulated genes (FBXW7 and FOXO3) in shMETTL13 (not affected by METTL3, METTL9 or METTL14 knockdown) compared to shCTRL. Significance was calculated using unpaired two-tailed t-test, results are displayed as TPM, mean ± SEM. C) Table of ALL patient characteristics and subgroups used for RNA-sequencing analysis (publicly available by the TARGET Initiative). Samples were divided into T-ALL (n=190) or B-ALL (n=78), diagnosis or relapse, as well as different high-risk factors (CNS infiltration, KMT2A-r and WBC). D) Expression levels of METTL3, METTL5, METTL9, METTL13 and METTL14 in T-ALL (n=162) and B-ALL (n=39) diagnosis samples (publicly available by the TARGET Initiative) compared to normal HSPCs (CD34+ CB, n=5) obtained through RNA-sequencing. Significance was calculated using multiple unpaired t-test, results are displayed as TPM, mean ± SEM. E) Expression levels of METTL3, METTL5, METTL9, METTL13 and METTL14 in T-ALL patient samples (publicly available by the TARGET Initiative) generated through RNA-sequencing, displayed as TPM, mean ± SEM. T-ALL samples were grouped by risk stratification, into standard risk (CNS negative, non KMT2A-r and low WBC, n=70), and high risk (CNS-infiltrated, KMT2A-r or high WBC, n=87). Significance was calculated using multiple unpaired t-test. F) Expression levels of METTL3, METTL5, METTL9, METTL13 and METTL14 in T-ALL patient samples (publicly available by the TARGET Initiative) generated through RNA-sequencing, displayed as TPM, mean ± SEM. T-ALL samples were grouped by disease stage, into diagnosis (standard risk, n=87) and relapse samples (BM relapse, n=5). Significance was calculated using multiple unpaired t-test. G) Survival probability in B-ALL patients samples (publicly available by the TARGET Initiative) by METTL13 expression levels (top and bottom 15%, n=6 in each group). Significance was calculated using Log-rank (Mantel-Cox) test. H) Correlation of METTL13 and TP53 as well as METTL13 and NOTCH1 in T-ALL patient samples, colored by the disease stage (diagnosis = blue, n=162 and relapse = red, n=18), results are displayed as TPM. Correlation was calculated using Pearson correlation coefficients with a two-tailed with 95% confidence interval.</p/> I) Correlation of METTL13 and TP53 in B-ALL patient samples, colored by disease stage (diagnosis = blue (n=39), relapse = red (n=39)), results are displayed as TPM. Correlation was calculated using Pearson correlation coefficients with a two-tailed with 95% confidence interval.

Our HSPC profiling after METTL knockdowns suggests a distinct role for METTL13 in leukemic transformation. To further investigate this, we focused on pediatric ALL. Indeed, many of the top mutated genes in pediatric T-ALL were downregulated following METTL3, METTL13 and METTL14 knockdown, such as TAL1, LEF1, PHF6, and PTEN (Fig. 5A) (62). Similarly, knockdown caused a loss of ETV6 and CREBBP, candidate driver genes in B-ALL (Fig. SF5A). In contrast, both CDKN2A, a gene with tumor suppressive potential in both T-ALL and B-ALL, as well as KRAS, a B-ALL driver gene, were upregulated (63) (Fig. SF5A). Knockdown of all three METTLs caused a decreased expression of stem cell surface marker CD34 (Fig. SF5A) (64). FBXW7 and FOXO3 were uniquely upregulated by the loss of METTL13, genes which have been identified as negative regulators of T-ALL progression, by inhibiting NOTCH1 activity and inducing apoptosis, respectively (Figure 5B) (65, 66).

To evaluate the role of METTL-family members in pediatric ALL progression, we performed RNA-sequencing analysis on 190 T-ALL and 78 B-ALL samples from the TARGET initiative (Fig. 5C, Supplemental table 3). We identified a significant loss of METTL3 in both T- and B-ALL samples compared to HSPCs (Fig. 5D). ALL samples were further grouped based on clinical risk factors, including white blood cell count (WBC), KMT2A-rearrangement (KMT2A-r) and central nervous system (CNS) infiltration (67). Among T-ALL samples, we identified an upregulation of METTL13 in both high risk (CNS-infiltrated, KMT2A-r or a WBC above 100) and relapsed samples (with bone-marrow origin) compared to standard risk diagnosis samples (CNS negative, non-KMT2A-r, and a WBC below 100) (Fig. 5E, 5F). This type of risk stratification was not observed for other METTL genes, including METTL3 or METTL14. Although no significant difference in METTL13 expression was identified between B-ALL samples, low METTL13 was associated with a higher probability of survival (Fig. 5G, SF5B). METTL13 expression correlated positively with TP53 expression in both B- and T-ALL, as well as with NOTCH1 in T-ALL (Fig. 5h, 5I). Our findings indicate converging functions of m6A writer genes METTL3 and METTL14, as well as METTL13 in pediatric ALL. In contrast, only METTL13 was associated with a high-risk profile in T-ALL and a decreased probability of survival in B-ALL.

### Loss of METTL13 impaired T-ALL proliferation and survival.

**Figure 6.**
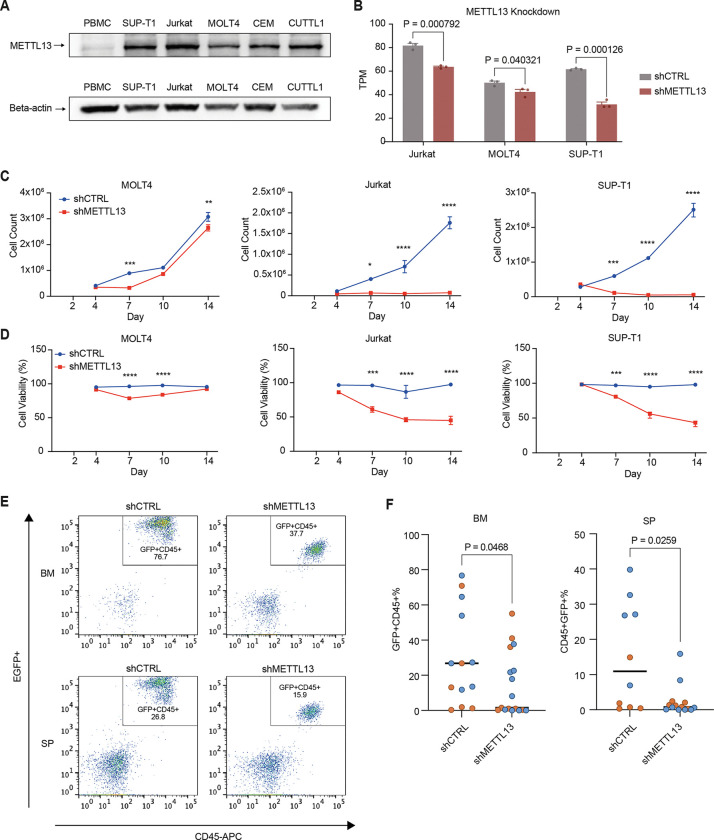
Loss of METTL13 impaired T-ALL cell proliferation and survival. A) Western blot image showing the expression level of METTL13 and beta-actin in normal PBMCs and T-ALL cell lines (SUP-T1, Jurkat, MOLT4, CEM and CUTTL1). B) Validation of METTL13 knockdown (shMETTL13) in T-ALL cell lines Jurkat (n=3), MOLT4 (n=3) and SUP-T1 (n=3) compared to the control (shCTRL, n=3 for each cell line) through RNA-sequencing. Significance was calculated using unpaired two-tailed t-test, results are displayed as TPM, mean ± SEM. C) Total number of viable cells following METTL13 knockdown in T-ALL cell lines MOLT4 (n=3), Jurkat (n=3) and SUP-T1 (n=3) from day 4 to day 14 post transduction compared to the control (n=3 for each cell line). Significance was calculated using two-way ANOVA. D) Cell viability (in percent) following METTL13 knockdown in T-ALL cell lines MOLT4 (n=3), Jurkat (n=3) and SUP-T1 (n=3) from day 4 to day 14 post transduction compared to the control (n=3 for each cell line). Significance was calculated using two-way ANOVA). E) Representative flow cytometry showing human EGFP^+^CD45^+^ leukemia engraftment in NSG-SGM3 mice transplanted with SUP-T1 cells. F) Engraftments of human EGFP^+^CD45^+^ were quantified by flow cytometry in bone marrow (BM) and spleen (SP) of SUP-T1 (blue) and MOLT4 (orange) transplanted mice (n = 10–17 mice per condition).

As the role and prognostic effect of METTL13 in pediatric leukemia is unexplored, we dived into the mechanistic link between METTL13 and leukemia propagation using T-ALL as a model. We first examined METTL13 expression in normal PBMCs and five T-ALL cell lines using western blot (Fig. 6A, SF6A, uncropped western blots). METTL13 was highly expressed in all T-ALL cell lines, with the highest expression in Jurkat, SUP-T1, and CUTTL1. To directly examine the function of METTL13, we knocked down METTL13 in three T-ALL cell lines with various level of METTL13 expression, which significantly reduced proliferation and survival starting from day 7 post-lentiviral transduction (Fig. 6B–D, SF6B). A stronger inhibition of cell viability and proliferation was found in high-METTL13-expressing cell lines (Jurkat and SUP-T1) compared to the relatively low-METTL13-expressing line (MOLT4), suggesting the cellular response may be dependent on cell-intrinsic expression levels.

To validate the *in vitro* results, we also performed an *in vivo* cell line-derived xenograft (CDX) assay. We transduced SUP-T1 and MOLT4 cells with EGFP^+^ scramble control shRNA or a shRNA targeting METTL13 to track METTL13 knockdown. EGFP+ cells were transplanted into NSG-SGM3 immuno-compromised mice. The leukemia engraftment was detected in both spleen and bone marrow and reached as high as 80% in the control bone marrow (Fig. 6E–F). METTL13 knockdown significantly reduced the leukemia engraftment rate in bone marrow (29.9% in control and 14.4% in shMETTL13) and spleen (15.6% in control and 2.4% in shMETTL13) (Fig. 6F, mean values). Thus, we confirmed that METTL13 is important for leukemia proliferation in both *in vitro* and *in vivo* T-ALL models.

### Knockdown of METTL13 promoted pathways inducing apoptosis and suppressing DNA synthesis in T-ALL cells.

We wanted to study aspects of METTL13 on a transcriptional level in T-ALL, therefore we performed RNA-sequencing on MOLT-4, Jurkat, and SUP-T1 cell lines following METTL13 knockdown. Our analysis revealed alterations to thousands of genes within each cell line (Fig. SF7A, Supplemental table 4). The three T-ALL cell lines clustered separately in PCA with most differentially expressed genes unique to each cell line, indicating a greater variability between the cell lines, which likely represents the heterogenicity of T-ALL (Fig. SF7B, SF7C). Despite this, all three cell lines also clustered into distinct METTL13 knockdown and control groups. The three cell lines shared only 1 529 significantly differentially expressed genes following METTL13 knockdown (Fig. SF7C-E), highlighting the importance of cell-specific m6A modifications.

**Figure 7.**
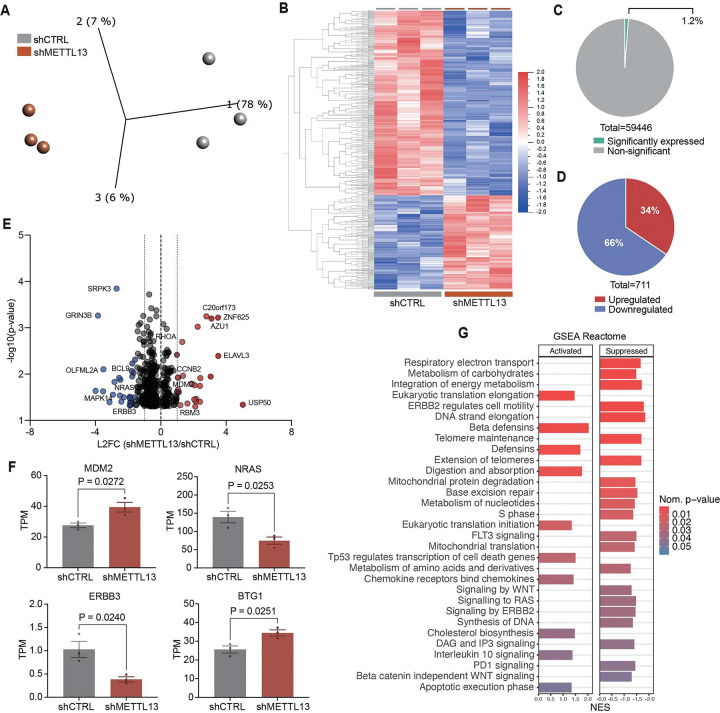
Knockdown of METTL13 promoted pathways inducing apoptosis and suppressing DNA synthesis in T-ALL cells. A) PCA plot of T-ALL cell lines as biological replicates following METTL13 knockdown (shMETTL13, n= 3) compared to the control (shCTRL, n=3). Significance was calculated using unpaired two-tailed t-test, p<0.05. B) Heat map of the top 400 differentially expressed genes looking at T-ALL cell lines as biological replicates in shMETTL13 (n=3) compared to the shCTRL (n=3). Significance was calculated using unpaired two-tailed t-test, p<0.05. C) Pie chart of the percentage of significantly expressed genes (p<0.05) in T-ALL cell lines as biological replicates (n=3) in shMETTL13 (n=3) compared to shCTRL (n=3). Significance was calculated using unpaired two-tailed t-test, p<0.05. D) Pie chart of the distribution of upregulated versus downregulated significantly expressed genes in T-ALL cell lines as biological replicates in shMETTL13 (n=3) compared to the shCTRL (n=3). Significance was calculated using unpaired two-tailed t-test, p<0.05. E) Volcano plot of significantly expressed genes (p<0.05) in T-ALL cell lines as biological replicates in shMETTL13 (n=3) compared to the shCTRL (n=3). Only protein-coding genes were included in this plot. Significance was calculated using unpaired two-tailed t-test, results are displayed as L2FC and negative log10 p-value (p <0.05). F) Dysregulated genes (MDM2, NRAS, ERBB3 and BTG1) in shMETTL13 (n=3) compared to shCTRL (n=3 Significance was calculated using unpaired two-tailed t-test, p<0.05, results are displayed as TPM, mean ± SEM. G) GSEA of some of the top significantly enriched Reactome pathways (Created in GSEA and MSigDB, nominal p-value <0.05) in shMETTL13 (n=3) compared to shCTRL (n=3) in T-ALL cells, using only protein-coding genes, displayed as activated (positive NES) or suppressed (negative NES).

To counteract the cell-intrinsic differences among cell types, the cell lines were treated as biological replicates, divided into shMETTL13 and shCTRL. PCA showed a separation between the two conditions, which was further emphasized by visualization of the gene expression pattern (Fig. 7A–B). Approximately 1% of all genes were differentially expressed, most being downregulated (Fig. 7C–D). We identified an upregulation of MDM2 and BTG1. BTG1 has been identified to induce cell cycle arrest and inhibit cell proliferation in several different malignancies (68). METTL13 knockdown inhibited B-ALL driver gene NRAS, and ERBB3, which is frequently overexpressed in cancer (Fig. 7E–F) (62, 69). Furthermore, GSEA revealed that knockdown of METTL13 suppressed nucleotide metabolism, WNT and RAS signaling, as well as DNA synthesis. Contrarily, METTL13 knockdown activated TP53 regulation of cell death genes, IL10 signaling and apoptotic execution phase. The results from the pathway analysis, together with both the *in vitro* and *in vivo* validation, highlight the potential impact METTL13 could have on leukemia cell survival and proliferation. Collectively, these data indicate a possible oncogenic role of METTL13 in pediatric T-ALL pathogenesis.

## Discussion

To better understand the role of m6A methylation and A-to-I editing, we investigated these RNA modifications in the context of pre-leukemic transformation and pediatric leukemia. We showed that ADAR1 had an overall inhibitory effect on the m6A complex in HSPCs yet promotes METTL13 expression. Our results uncover a novel role for METTL13 in regulating gene expression in HSPCs, both overlapping with the m6A writer complex, and with unique effects on genes involved in apoptosis and cancer progression. We have also identified that METTL13 regulates T-ALL cell survival and proliferation both *in vitro* and *in vivo* and was associated with a high-risk profile in pediatric T-ALL.

Previous studies have shown that RNA methylation by the m6A complex induced RNA editing, by increasing ADAR1 levels in response to IFN stimulation (55), and METTL3 was found to increase protein levels of ADAR1 in glioblastoma (70). In contrast, ADAR1 was found to preferentially bind to m6A-depleted RNA transcripts, indicating a negative correlation between A-to-I editing and m6A (71). We revealed a general suppressive effect of ADAR1 on members of the m6A complex, on a transcriptional level in human HSPCs. Overexpression of ADAR1 inhibited the main writer genes METTL3 and METTL14, as well as erasers FTO and ALKBH5. In contrast, an orphan METTL-family member, METTL13, was augmented by the induction of ADAR1. Whether METTL13 acts in co-ordinance with other METTL-family members, or has similar effects on HSC maintenance as ADAR1, will need to be carefully investigated by future studies.

Aberrant activity of m6A modifications have been shown to promote the development of both solid tumors and hematological malignancies (41). Despite this, the distinct role of each METTL-family member remains undefined. Therefore, we decided to knockdown m6A writers METTL3 and METTL14, and less studied METTL5, METTL9 and METTL13 in HSPCs, individually. Knockdown revealed that many differentially expressed genes were regulated both by the m6A writer complex and METTL13, suggesting that METTL13 may be acting in convergence with the m6A writer complex. The overlapping genes include key oncogenic genes, such as TP53, MDM2, and c-MYC. GSEA revealed many commonly altered pathways, mainly related to inflammatory signaling. GSEA also identified numerous pathways uniquely altered by METTL13 knockdown, including p53 signaling and several cancer-associated pathways

Recent reports have identified METTL13 as a lysine methyltransferase that modifies the eEF1a protein at lysine 55, thus promoting translation (72, 73). METTL13 has been correlated with a decreased survival probability in malignancies such as lung and pancreatic cancer and to augment metastasis in gastric cancer (73, 74). Existing studies focus mainly on methylation of eEF1A and changes in translational output, several highlighting the potential oncogenic role of METTL13 in cancer. In contrast, loss of METTL13 was considered a poor prognostic marker in clear cell renal cell carcinoma, inhibiting proliferation and metastatic capacity of cancer cells (75). However, research on METTL13 and its role in HSC biology and leukemia is sparse. The extensively overlapping gene expression programs between METTL3-METTL14 complex and METTL13 identified in this study indicate that functions of METTL13 align with the m6A pathway in the cellular context of HSCs, which is surprising since METTL13 was recently reported as an eEF1a methyltransferase. Future studies are necessary to systematically de-couple the roles of METTL13 in m6A regulation and protein translation control.

Our findings highlight the effects of METTL13 in malignant transformation, which prompted further analysis to unravel its specific role in hematological diseases. We examined transcriptional alterations to candidate driver genes in pediatric ALL following knockdown of members of the METTL-family in HSPCs. We identified a loss of several critical oncogenic drivers of both B- and T-ALL following knockdown of METTL3, METTL13 and METTL14, including TAL1, LEF1 and CREBBP, as well as an upregulation of tumor suppressor CDKN2A. To further explore the role of the METTL-genes in pediatric ALL, we utilized an RNA-sequencing dataset from the TARGET initiative, and report that METTL13 was overexpressed in both high-risk and relapsed T-ALL. Finally, we report reduced survival and proliferation of T-ALL cells following METTL13 knockdown, both *in vitro* and *in vivo*. Knockdown of METTL13 significantly reduced the leukemia engraftment rate in the CDX model. RNA-sequencing analysis of T-ALL cells with depleted METTL13 expression revealed activation of p53 signaling and apoptosis, while inhibiting DNA synthesis and signaling pathways crucial for cell proliferation and leukemic progression, such as WNT and RAS signaling.

In conclusion, the collective findings following METTL13 knockdown in HSPCs and T-ALL cells suggest that METTL13 may be involved in promoting pre-leukemic development of HSPCs and in the pathogenesis of T-ALL. Future studies are necessary to functionally determine effects of METTL13 in leukemic progression, yet our findings highlight a novel, distinct role of METTL13 in T-ALL development.

## Supplementary Material

Supplemental Figures

Supplemental Figure 1.

A) Validation of ADAR1 overexpression in human CD34^+^ HSPCs (n=3) compared to the backbone control (pCDH, n=3). Significance was calculated using unpaired two-tailed t-test, results are displayed as TPM, mean ± SEM.

B) Expression levels of genes in the METTL-family following ADAR1 overexpression compared to the control. Significance was calculated using unpaired two-tailed t-test, results are displayed as TPM, mean ± SEM.

Supplemental Figure 2.

A) Validation of METTL knockdown (shMETTL3 (n=3), sMETTL5 (n=3), shMETTL9 (n=3), shMETTL13 (n=3) and shMETTL14 (n=3)) in human CD34^+^ HSPCs compared to the control (shCTRL, n=3) through RT-qPCR. Significance was calculated using unpaired two-tailed t-test, results are displayed as expression level relative to the housekeeping gene (HPRT), mean ± SEM.

B) Validation of METTL knockdown (shMETTL3 (n=4), sMETTL5 (n=2), shMETTL9 (n=3), shMETTL13 (n=4) and shMETTL14 (n=3)) in HSPCs compared to the control (shCTRL, n=5) through RNA-sequencing. Significance was calculated using unpaired two-tailed t-test, results are displayed as TPM, mean ± SEM.

C) PCA plot of shMETTL3, shMETTL5, shMETTL9, shMETTL13 and shMETTL14 compared to shCTRL with the outliers labeled (excluded in sequential analysis). Created in Qlucore Omics Explorer.

Supplemental Figure 3.

A) Venn diagram of GSEA Wiki Pathways between shMETTL3, shMETTL9, shMETTL13 and shMETTL14 in human CD34^+^ HSPCs compared to shCTRL (FDR q<0.1).

B) Venn diagram of GSEA Reactome pathways between shMETTL3 (n=4), shMETTL9 (n=3), shMETTL13 (n=4) and shMETTL14 (n=3) compared to shCTRL (n=5) (FDR q<0.1).

C) Venn diagram of GSEA KEGG pathways between shMETTL3, shMETTL9, shMETTL13 and shMETTL14 compared to shCTRL (FDR q<0.1).

D) Bar plot of all unique GSEA Wiki Pathways (FDR.q <0.1) in shMETTL13 compared to shCTRL. Results are displayed by NES.

Supplemental Figure 4.

A) Top differentially expressed genes following METTL13 (shMETTL13, n=4) knockdown in human CD34^+^ HSPCs compared to control (shCTRL, n=5). Significance was calculated using unpaired two-tailed t-test (p < 0.05), results are displayed as L2FC (shMETTL13/shCTRL).

B) ORA of top disease ontologies in shMETTL13 compared to shCTRL. Created in R, packages ClusterProfiler, DOSE and Enrichplot, with statistics set to: q.value ≤ 0.1, L2FC cutoff = 1, p-adjust method = BH.

Supplemental Figure 5.

A) Dysregulated genes (ETV6, CREBBP, KRAS, CD34 and CDKN2A) METTL3 (shMETTL3, n=4), METTL9, (shMETTL9, n=3) METTL13 (shMETTL13, n=4) and METTL14 (shMETTL14, n=3) knockdown in human CD34^+^ HSPCs compared to the control (shCTRL, n=5). Significance was calculated by ordinary one-way ANOVA with multiple comparisons compared to shCTRL, as well as Dunnett correction, results are displayed as TPM, mean ± SEM.

B) Expression levels of METTL3, METTL5, METTL9, METTL13 and METTL14 in B-ALL patient samples (publicly available by the TARGET Initiative) generated through RNA-sequencing. B-ALL samples were grouped by disease stage, into diagnosis (n=39) or relapse samples (n=39), samples are longitudinal. Significance was calculated using multiple unpaired t-test, results are displayed as TPM, mean ± SEM.

Supplemental Figure 6.

A) Full length western blot image showing the expression level of METTL13 and beta-actin in normal PBMCs and T-ALL cell lines (SUP-T1, Jurkat, MOLT4, CEM and CUTTL1).

B) Validation of METTL13 knockdown (shMETTL13) in T-ALL cell lines Jurkat (n=1), MOLT4 (n=1) and SUP-T1 (n=1) compared to the control (shCTRL, n=1 for each cell line) through RT-qPCR. Significance was calculated using unpaired two-tailed t-test, results are displayed as expression level relative to the housekeeping gene (HPRT), mean ± SEM.

Supplemental Figure 7.

A) Distribution of differentially expressed genes in shMETTL13 compared to shCTRL in each T-ALL cell line separately (n=3 for each cell line and condition). Significance was calculated using unpaired two-tailed t-test, p < 0.05.

B) PCA plot of shMETTL13 compared to shCTRL in T-ALL cell lines Jurkat, MOLT4 and SUP-T1 (n=3 for each cell line and condition). Created in Qlucore Omics Explorer.

C) Venn diagram of all differentially expressed genes (p<0.05) in shMETTL13 compared to shCTRL in each T-ALL cell line separately (n=3 for each cell line and condition). Significance was calculated using unpaired two-tailed t-test, p < 0.05.

D) Venn diagram of significantly upregulated genes (p<0.05) in shMETTL13 compared to shCTRL in each T-ALL cell line separately (n=3 for each cell line and condition). Significance was calculated using unpaired two-tailed t-test, p < 0.05.

E) Venn diagram of significantly downregulated genes (p<0.05) in shMETTL13 compared to shCTRL in each T-ALL cell line separately (n=3 for each cell line and condition). Significance was calculated using unpaired two-tailed t-test, p < 0.05.

Supplemental Tables

**Supplemental Table 1.** Differentially expressed genes following ADAR1 overexpression in HSPCs

**Supplemental Table 2.** Differentially expressed genes following METTL3, METTL5, METTL9, METTL13 and METTL14 knockdown in HSPCs

**Supplemental Table 3.** Clinical characteristics of ALL patient samples provided by TARGET

**Supplemental Table 4.** Differentially expressed genes following METTL13 knockdown in T-ALL cell lines

**Supplemental Table 5.** RT-qPCR Primers

Supplementary Files

This is a list of supplementary files associated with this preprint. Click to download.

• Supplementalfigure1.png

• Supplementaltable5.xls

• Supplementalfigure6.png

• Supplementalfigure7.png

• Supplementaltable3.xls

• Supplementaltable1.xls

• Supplementalfigure5.png

• Supplementalfigure3.png

• Supplementaltable4.xls

• Supplementalfigure4.png

• Supplementalfigure2.png

• Supplementaltable2.xls

• uncroppedwesternblots.png

## Data Availability

The RNA-sequencing dataset used in this study will be uploaded to an appropriate data portal upon request with a special password for editors and reviewers. The data will be made publicly available upon acceptance for publication. Further information and requests for resources and reagents should be directed to and will be fulfilled by Dr. Frida Holm (frida.holm@ki.se).
